# Human Papillomaviruses; Epithelial Tropisms, and the Development of Neoplasia

**DOI:** 10.3390/v7072802

**Published:** 2015-07-16

**Authors:** Nagayasu Egawa, Kiyofumi Egawa, Heather Griffin, John Doorbar

**Affiliations:** 1 Division of Virology, Department of Pathology, University of Cambridge, Tennis Court Road, Cambridge CB2 1QP, UK; E-Mails: ne259@cam.ac.uk (N.E.); hmg36@cam.ac.uk (H.G.); 2 The Francis Crick Institute Mill Hill Laboratory, the Ridgeway, Mill Hill, London NW7 1AA, UK; 3 Department of Dermatology, The Jikei University School of Medicine, 3-25-8 Nishi-shinbashi, Minato-ku, Tokyo 105-0003, Japan; E-Mail: amamino.kuroibo@gmail.com; 4 Department of Microbiology, Kitasato University School of Allied Health and Sciences, 1-15-1 Kitasato, Sagamihara, Kanagawa 252-0373, Japan

**Keywords:** human papillomavirus, tropism, diversity, evolution, tissue stem cells, niche, carcinogenesis

## Abstract

Papillomaviruses have evolved over many millions of years to propagate themselves at specific epithelial niches in a range of different host species. This has led to the great diversity of papillomaviruses that now exist, and to the appearance of distinct strategies for epithelial persistence. Many papillomaviruses minimise the risk of immune clearance by causing chronic asymptomatic infections, accompanied by long-term virion-production with only limited viral gene expression. Such lesions are typical of those caused by Beta HPV types in the general population, with viral activity being suppressed by host immunity. A second strategy requires the evolution of sophisticated immune evasion mechanisms, and allows some HPV types to cause prominent and persistent papillomas, even in immune competent individuals. Some Alphapapillomavirus types have evolved this strategy, including those that cause genital warts in young adults or common warts in children. These strategies reflect broad differences in virus protein function as well as differences in patterns of viral gene expression, with genotype-specific associations underlying the recent introduction of DNA testing, and also the introduction of vaccines to protect against cervical cancer. Interestingly, it appears that cellular environment and the site of infection affect viral pathogenicity by modulating viral gene expression. With the high-risk HPV gene products, changes in E6 and E7 expression are thought to account for the development of neoplasias at the endocervix, the anal and cervical transformation zones, and the tonsilar crypts and other oropharyngeal sites. A detailed analysis of site-specific patterns of gene expression and gene function is now prompted.

## 1. Introduction

**Figure 1:**
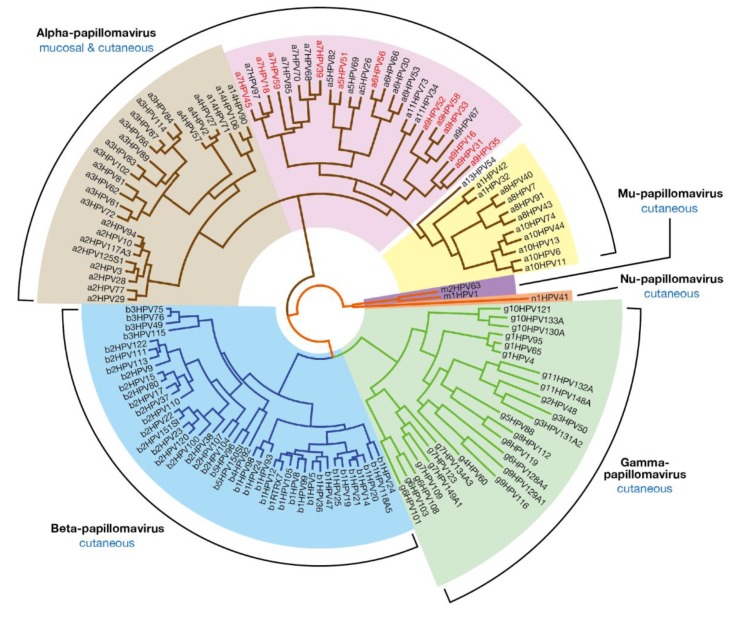
Evolutionary Relationship between Human Papillomaviruses. The human papillomaviruses types found in humans fall into five genera, with the Alpha-, Beta- (**blue**) and Gammapapillomavirus (**green**) representing the largest groups; Human papillomaviruses types from the Alphapapillomavirus genus are often classified as low-risk cutaneous (**light brown**); low-risk mucosal (**yellow**); or high-risk (**pink**) according to their association with the development of cancer. The high-risk types highlighted with red text are confirmed as “human carcinogens” on the basis of epidemiological data. The remaining high-risk types are “probable” or “possible” carcinogens. Although the predominant tissue associations of each genus are listed as either cutaneous or mucosal, these designations do not necessarily hold true for every member of the genus. The evolutionary tree is based on alignment of the *E1*, *E2*, *L1*, and *L2* genes [26]. HPV sequence data was be obtained from PaVE [1].

Papillomaviruses have been discovered in a wide array of vertebrates. More than 300 papillomaviruses have been identified and completely sequenced, including over 200 human papillomaviruses (PaVE: Papillomavirus Episteme [1]). One of the most distinctive characteristics of the papillomavirus group is their genotype-specific host-restriction, and the preference of particular papillomavirus types for distinct anatomical sites, where they cause lesions with distinctive clinical pathologies [2]. These include benign hyper-proliferative lesions such as warts, as well as unapparent or asymptomatic precursor lesions, that can in some instances progress to high-grade neoplasia and invasive malignant cancer. The association of “high-risk” human papillomavirus (HPV) types (see legend to Figure 1) with cervical cancer is now well established, and provides a rationale for the introduction of HPV DNA testing in cervical screening, as well as the development of prophylactic vaccines against HPV16 and 18 which are the major papillomavirus types responsible for cervical cancer. Such typing studies have also revealed a plethora of HPV types, both high and low-risk, in oral mouthwash samples despite the absence of apparent clinical disease [3], as well as in skin swabs and plucked hairs taken from immunocompetent individuals [4,5,6,7,8,9]. The predominant asymptomatic skin HPV types come primarily from the genera Betapapillomavirus and Gammapapillomavirus, and at a population level, members of these genera are very successful, infecting children at a young age to produce persistent subclinical infections [4]. Changes in the epithelial micro-environment, as can occur following immunosuppression or in individuals suffering from epidermodysplasia verruciformis (EV), can allow these HPV types to produce visible papillomas, and in some situations can facilitate the development of cancers [10]. Certain Beta HPV types are a significant cause of non-Melanoma skin cancer in susceptible individuals, although the molecular mechanism by which they facilitate cancer progression appears to be somewhat different from what has been worked out for the Alphapapillomavirus types [11]. When considered together, it appears that different papillomavirus types have evolved distinct life-cycle strategies, which allow them to thrive and produce viral progeny at different epithelial sites. At least part of this variation reflects the different ability of papillomaviruses to interact with the immune system, and to produce productive infections that are visible at the macroscopic level. The complex immune evasion strategies that underlie the ability to produce such lesions are a particular characteristic of the Genus Alphapapillomavirus, with low-risk Alphapapillomavirus types in particular (Figure 1), often being responsible for recalcitrant warts even in immunocompetent hosts [12]. By contrast, most members of the genera β- and Gammapapillomavirus only cause such visible lesions when the normal immune response of the host is compromised [13,14,15,16]. The clinical importance of papillomaviruses, and their relatively small genomic size, has led to many full genomic sequences becoming available in recent years. The availability of such extensive sequence information, combined with a developing clinical and biochemical understanding of disease-biology, means that papillomaviruses are an ideal model system to understand how evolution can influence viral tropisms, pathogenicity, and the underlying molecular processes that govern disease outcome [17,18,19,20]. As discussed below, this “natural experiment” in infection is also providing us with insight into epithelial cell biology and immunology.

## 2. Papillomavirus Diversity at the Level of Genotype, Epithelial Tropism and Pathogenicity

Papillomaviruses comprise a diverse group of viruses that infect both humans and animals. Their origin is linked to changes in the epithelium of their ancestral host that occurred at least 350 million years ago. Since then, they have co-evolved as their different host species have evolved, with little cross-transfer between species [17,18]. They are now found in birds, reptiles, marsupials and other mammals, pointing to an earlier evolutionary appearance than was initially suspected. Recent phylogenetic analysis suggests, however, that the “generalist” ancestral papillomavirus may not have followed an identical evolutionary path to that of their hosts, but paralleled the evolution of host resources or attributes, such as the presence or absence of fur or the evolution of sweat glands. These host-adaptations are thought to have created new ecological niches for papillomavirus to colonise, which in turn drove viral diversity followed by co-speciation with their hosts [17]. Through this route, papillomaviruses have developed their remarkable species specificity as well as a great diversity of epithelial tropisms. With over 240 distinct papillomavirus types classified into 37 genera, papillomavirus may perhaps be considered as one of the most successful families of vertebrate viruses [17,21,22].

The classification of papillomaviruses is based on nucleotide sequence comparison rather than on serology, with individual HPVs being referred to as genotypes [22]. Data from vaccine trials has shown that there is limited antibody cross-reactivity, except between closely related genotypes [23], with the major coat protein of the virus (L1) containing hypervariable loops that are exposed on the virion surface [24,25]. The L1 gene was chosen early on as the standard for PV classification, and for some papillomavirus types, there is little sequence information outside of this region. To be classified as distinct types, individual papillomaviruses must be at least 10% divergent from each other in their L1 nucleotide sequence. These papillomavirus “types” are grouped into larger phylogenetic groupings or genera, which are categorised with a Greek letter followed by a number that indicates the species (Figure 1) [22].

Thus the species Alphapapillomavirus 9 includes HPV types 16, 31, 35, 33, 52, 58 and 67. Ideally, papillomavirus (PV) classification should integrate phylogeny, genome organization, biology and pathogenicity as a single property, rather than being based simply on genomic sequence analysis, or the analysis of genomic fragments [21]. This phylogenetic species concept, although potentially useful, is complex to implement, however, and for many papillomavirus types, detailed information regarding their biology is only poorly defined.

In general, sequence-based phylogeny does provide some useful insight into disease association, although closely related types can in some instances show distinct pathologies. HPV4, 65 and 95 are encompassed in the Gammapapillomavirus 1 species, for instance, but while HPV4 and 65 induce indistinguishable pigmented wart-like lesions mainly on the palmoplantar or lateral surface of hands and feet [27,28,29], HPV95 induces less obvious unpigmented papules primarily on plantar epithelial surfaces [30]. HPV6 and 11 are similarly contained within a common species-grouping (Alphapapillomavirus 10), and although both cause papillomas of similar appearance, HPV6 shows a marked predilection for genital sites, when compared to HPV11, which is the most prominent type at oral sites [31,32,33,34]. A third example comes from the Alphapapillomavirus 8 species which includes HPV40, which causes mucosal lesions, and HPV7, which is the cause of “butchers” warts that develop at cutaneous sites, particularly the hands [35]. Explaining such subtle tropism differences is beyond our current understanding of virus biology, and is not of obvious medical importance. It is however likely that such tropism differences reflect differences in viral gene function, patterns of gene expression and epithelial regulation at different body sites. Perhaps of greater importance are the differences in cancer risk associated with the high-risk types. HPV16, 31 and 35 are all contained within the Alphapapillomavirus 9 species group and are classified as “carcinogenic to humans” by The International Agency for the Research on Cancer (IARC) [36]. The association between HPV16 and cervical cancer is more than 10 times stronger than that between either HPV31 or HPV35 however [37], and of these three types, HPV16 is uniquely associated with tumours of the oropharyngeal region [38]. To explain this will require a dissection of virus-specific gene expression at this particular epithelial site, and an understanding of virus protein function and the extent to which proteins encoded by different HPV types affect common molecular pathways.

**Figure 2:**
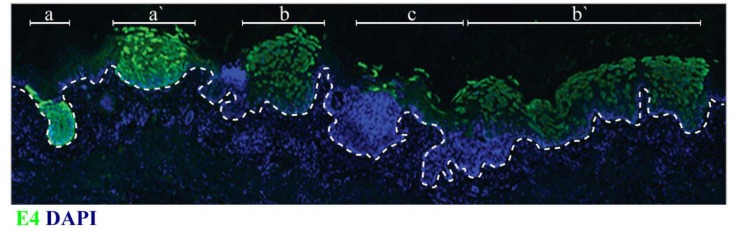
Viral gene-expression in adjacent Betapapillomavirus lesions. Immunostaining for the Betapapillomavirus E4 protein (**green**) reveals distinct patterns of expression in different lesions in an immunosuppressed individual. Lesion (**a**) and (**a`**) are atypical, and show marked basal and suprabasal staining; This pattern of E4 expression is distinct from typical E4 pattern of expression, which is usually restricted to the mid/upper epithelial layers as seen in region (**c**); Lesions (**b**) and (**b`**) show an intermediate pattern of E4 staining with expression close to the basal layer, as well as in the suprabasal cell layers (see also review [11]).

From the above, it is apparent that viral pathogenicity depends on multiple factors, including the virus genotype, the nature of the cell infected (tropism) and the status of host immunity. Our current thinking suggest that tropism is controlled primarily at the level of viral gene expression rather than at the level of viral entry into the cell [39], and that regulatory elements within the long control region (LCR) are of key importance in determining the tissue range of different HPV types [40,41]. Differences in infectivity may however be a contributing factor, and it has been suggested that this may correlate with differences in surface charge distribution between cutaneous and mucosal virions [42]. Although the diseases caused by specific HPV types sometimes occur at non-typical sites, this is uncommon, with such lesions often exhibiting non-typical morphology and pathology [43]. The idea that papillomaviruses have epithelial sites where they have evolved to complete their productive life-cycle, and epithelial sites where they do this less-well or not at all, makes good sense when we consider the small number of instances where cross-species infection has been documented. Bovine Papillomavirus type 1 infection of cattle leads to the development of benign cutaneous lesions that may regress, whereas infection of horses typically results in the development of locally aggressive, non-regressing equine sarcoids that are non-permissive for virus production [44,45,46,47]. In this case, it appears that the BPV E5 gene, which is important for the production of fibropapillomas in cattle, can stimulate aberrant cell proliferation in the dermal layers in the horse [47]. Although cottontail rabbit papillomavirus (CRPV) is not a fibropapillomavirus like BPV, a similar concept explains its ability to generate non-productive lesions in domestic rabbits, that have a tendency to develop over time into neoplasia and cancer [48,49]. In this case, it is the E6 and E7 proteins, along with the viral transcription factor E2, that are ultimately important for the development of the cancer phenotype [50,51]. Historically, these ideas are not new to the field of tumour virus biology, with a large body of experimental work using animal models to understand how members of the polyoma and adenoviruses families can cause tumours [52,53]. In the case of adenoviruses, it is the E1A and E1B proteins, and with polyomaviruses it is the T antigens, that when deregulated can cause tumours in experimental systems [54,55], and it is now well known that these proteins share functional similarity with the E6 and E7 proteins of the high-risk papillomaviruses [56,57]. In fact, our current knowledge suggests that it is the deregulated expression of the high-risk E6 and E7 proteins that leads to the development of neoplasia at specific epithelial sites, and that these infections should be regarded as “non-productive or abortive infections” rather than “ordered” productive infections, where the level and pattern of viral gene expression is properly controlled [58,59]. It has been reported that several factors such as the type of cell, hormone as well as inflammatory cytokines control viral gene expression [41,60,61,62]. With this in mind, it is clearly important to understand how epithelial environment, cell type and host immunity act together to drive neoplasia, and we suspect that this will be an important topic of future research. Although this line of thinking fits well with our understanding of how high-risk HPV types cause neoplasia and cancer at specific sites, including the cervix, anus and oropharynx, the same broad principles also explain the elevated risk of non-melanoma skin cancer posed by Betapapillomaviruses, even though the molecular detail of how these viruses cause cancer is different [11]. Betapapillomavirus types are prevalent as asymptomatic infections in normal skin and mucosa in the general population [3,63,64], with infection occurring soon after birth [9]. In immunocompromised individuals, including those suffering from epidermodysplasia verruciformis, it appears that normal patterns of viral gene expression can become deregulated, allowing a level of viral gene expression that would not be tolerated in immune competent individuals (Figure 2) [11].

Interestingly, we have also seen that certain papillomaviruses, which are usually associated with symptomatic lesions following infection, can persist at the site of infection following immune regression, with reactivation occurring as the immune environment changes [65,66]. For both α- and Betapapillomavirus types, this work suggests that the immune system provides an important check on the extent of viral gene expression, and that overexpression of viral gene products can predispose to cancer progression following infection by certain β HPV types as well as for the high-risk Alphapapillomaviruses. The existence of latent papillomavirus infections remains to be conclusively validated in humans, but experiments in animal models suggest that this is likely [67,68].

## 3. Genome Structure and the Classification of Viral Gene Products

**Figure 3:**
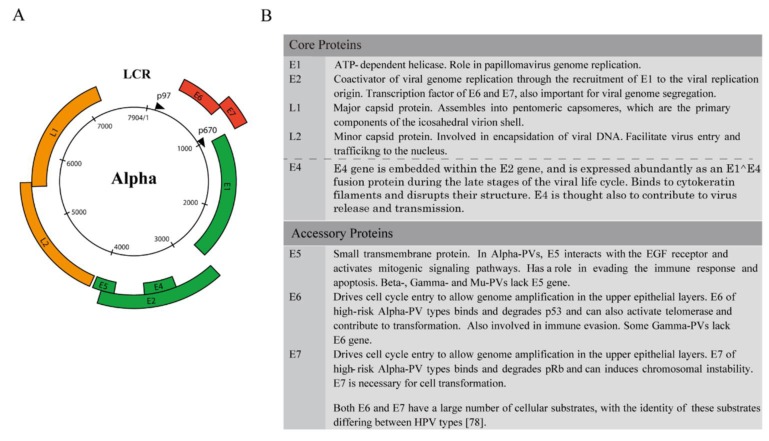
Alphapapillomavirus genome organization and the function of HPV proteins. (**A**) Genome organization typical of the high-risk Alphapapillomavirus types is illustrated by the genome of HPV16. The early (p97) and late (p670) promoters are marked by arrows. The six early ORFs (E1, E2, E4 and E5 (**in green**) and E6 and E7 (**in red**)) are expressed from the different promoters at different stages during epithelial cell differentiation. The late ORFs (L1 and L2 (**in yellow**)) are expressed from the p670 promoter in the upper epithelial layers as result of changes in splicing. The LCR/URR also contains the replication origin as well as post-transcriptional control sequences that contribute to viral gene expression. (**B**) The function of viral proteins. All known papillomavirus encodes a group of “core” proteins that were present early on during papillomavirus evolution, and which are conserved in sequence and in function between PV types. These include E1, E2, L2 and L1. The E4 protein may also be a core protein that has evolved to meet papillomaviruses epithelial specialization. The accessory proteins have evolved in each papillomavirus type during adaptation to different epithelial niches. The sequence and function of these genes are divergent between types. In general, these proteins are involved in modifying the cellular environment to facilitate virus life cycle completion, contributing to the virulence and pathogenicity. Knowledge of accessory protein function comes primarily from the study of Alphapapillomavirus types.

As outlined above, papillomaviruses can complete their life-cycle at many different anatomical sites, including the skin, anogenital tract and the oral cavity, but do not always produce clinically apparent lesions, sometimes persisting in the epithelium as non-productive latent infections. To progress beyond these basic observations requires an understanding of viral protein function and the expression patterns of the viral gene products in the epithelium. The viral genome is a circular double-stranded DNA episome, which ranges in length from 6953 bp for Cheloniamydas papillomavirus type 1 (CmPV1) to 8607 bp for Canine papillomavirus type 1 (CPV1). Viral genomes generally contain one regulatory region designated as the URR (upstream regulatory region) or LCR (long control region), which contains transcription factor-binding sites and the replication origin, and two groups of ORFs, which are designated early (E) or late (L) [69]. Some papillomavirus types, such as CPV, contain a second regulatory region between the end of the early region and the start of the late regions [70,71]. Despite variation in the size and number of ORFs, all PV members include a URR, along with a group of proteins with a high level of conservation that are found in all sequenced papillomavirus types (Figure 3A).

These “core” genes include the E1 and E2 proteins necessary for viral replication [72,73], and the viral late proteins L1 and L2 [24,74]. The remaining viral genes (E6, E7, E5) may be considered as “accessory” genes that have evolved to facilitate replication in stratified epithelium (Figure 3B) [56,57,75]. These early gene products are generally more divergent than E1 and E2, both functionally and at the primary amino acid sequence level, and are not universally present in all papillomavirus types. Interestingly, the viral E4 protein has some characteristics of a core protein, in that it is present in most if not all papillomaviruses, but is also variable at the primary amino acid sequence level, which at first site could suggest a divergent function [76]. It is thought however that E4 plays a key role in virus escape from the cornified epithelial layers, and that evolution has driven E4 divergence to allow it to carry out its common function at different anatomical sites where epithelial structure differs. In general, the viral core genes are thought to have existed early during papillomavirus evolution, and carry out essential functions during the virus life cycle in the epithelium. L1 encodes primary structural protein in the virus capsid [77], with the minor capsid protein L2 binding to the circular viral DNA to facilitate optimal genome encapsidation [78]. E1 encodes a virus-specific DNA helicase [72], while E2 functions in viral transcription, replication and genome partitioning [73]. The remaining genes (E6, E7 and E5) encode proteins that modify the cellular environment, and in many cases perform similar but not necessarily identical functions during the life cycle of different papillomaviruses to support the production of progeny virions and to affect virulence. As such, it is thought that a major determinant of virus tropism may lie in the function of these accessory genes as well as in their regulation.

## 4. Productive Papillomavirus Life Cycle and Non-Productive Infection

Because the study of papillomaviruses has been driven by the association of the high-risk Alphapapillomavirus types with anogenital and oropharyngeal cancers, our knowledge of these types is perhaps more complete. However, there are some general principles that extend across the different genera that are worth overviewing before commenting further on the specific characteristics of different papillomavirus groups [79]. It is widely accepted that papillomavirus infection occurs once virus particles gain access to the epithelial basal cells or stem cells, which in many cases involves some level of epithelial trauma. In all cases, it is thought that virions bind initially to the glycosaminoglycan (GAG) chains of heparan sulfate proteoglycan (HSPG) in a charge-based manner, and that this leads to conformational changes in the virion which expose a furin/proprotein convertase cleavage site at the amino terminus of L2. It is generally thought that virus entry requires interaction with a secondary receptor, which still remains to be properly characterised [39]. Importantly, these early HSPG-dependent events have been shown to occur on the extracellular basement membrane (BM) in the murine cervico-vaginal model of HPV16 infection [80], close to where the target basal cells reside.

**Figure 4:**
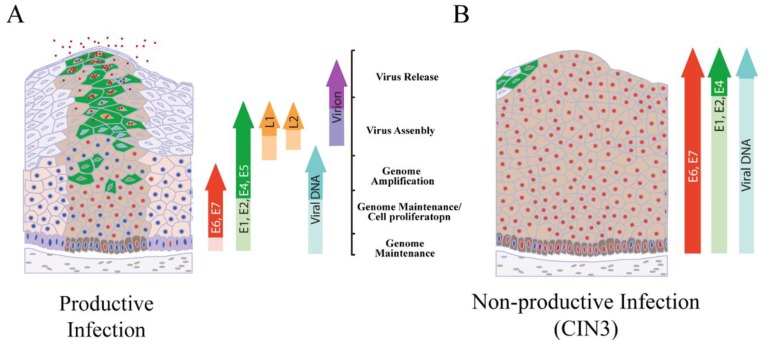
Regulation and deregulation of the high-risk Alphapapillomavirus life cycle. (**A**) The papillomavirus life cycle is regulated during epithelial cell differentiation and is shown diagrammatically. Cells that are driven through the cell cycle as a result of E6 and E7 expression are marked with red nuclei. The up-regulation of viral proteins necessary for genome amplification (*i.e.*, E1 and E2) requires activation of the viral late promoter in the upper epithelial layers (cells shown in green with red nuclei), with virus particles subsequently being released from the epithelial surface; (**B**) In HPV-associated neoplasia, late gene expression is retarded, and although the order of events remains the same, the production of infectious virions is restricted to smaller and smaller areas close to the epithelial surface. This situation is thought to be accompanied elevated E6/E7 expression, and represents a non-productive or poorly productive abortive infection. Integration of HPV DNA into the host cell genome is facilitated by deregulated E6/E7 expression. If integration disrupts the E1/E2 region this can allow the persistent high-level expression of E6 and E7 and the accumulation of genetic errors in the host genome. Eventually, the productive virus life cycle is no longer supported and viral episomes are lost (reviewed in [83]).

After PV infection, an initial phase of genome amplification takes place prior to maintenance of the viral episome at low copy number in the infected basal cells (Figure 4A) [81,82].

The viral replication proteins E1 and E2 are thought to be essential for this initial amplification, but E1 may be dispensable for maintenance-replication once copy number has stabilised at 50–100 [65]. E2 has an established role in genome partitioning, replication and viral gene expression. Experimental systems have shown at least a two-log increase in viral copy number during genome amplification [65], with E1, E2 and cellular DNA replication proteins being crucial for viral genome amplification *in vitro* [72,73,84]. The extent of genome amplification may be higher (four-log increase) during infection *in vivo*, as suggested from laser capture analysis of productive infections in rabbits [65]. The essential role of E6 and E7 in the viral lifecycle is primarily to modify the cellular environment to allow viral genome amplification, mainly by driving S-phase re-entry in the upper epithelial layers. Genome amplification occurs as the infected cell moves from an S to a G2-like phase before committing to full differentiation. In the case of high-risk types, E6 and E7 appear also to drive cell proliferation in the basal and parabasal layers [83]. By contrast, in the case of low-risk types (HPV6, HPV11 or other HPV types that have a tendency to cause benign lesions), the precise role of these proteins in the infected basal cells is unclear. In fact, functional differences in E6 and E7 represent a major determinant of HPV disease pathogenicity between HPV types [85]. During the virus life cycle, E5 also contributes to viral genome amplification as a result of its ability to stabilize epidermal growth factor (EGF) receptor and to enhance mitogen-activated protein (MAP) kinase activity. E5 also modulates both extracellular-signal-regulated kinase 1/2 (ERK 1/2) and p38 independently of EGF receptor [75]. Interestingly, the nuclear localisation and export signals in E1 are phosphorylated by these MAP kinases, which in turn enhances the nuclear localisation of E1 protein which is necessary for genome amplification [86,87]. In the upper layers of the epithelium, amplified viral genomes are packaged into virus particles produced from the major (L1) and minor (L2) virus coat proteins. The E4 protein, which accumulates at very high levels in cells supporting virus synthesis [76], appears to have a primary function in virus release and/or transmission, but acts also to optimise the success of virus genome amplification. In high-risk HPV types, the E4 protein assembles into amyloid fibrils that can disrupt keratin structure and compromise the normal assembly of the cornified envelope [88,89]. Although not yet precisely defined, E4 amyloid fibres may contribute to virion release from the upper epithelial layers, and therefore infectivity and transmission.

Although high-risk HPV infection is common, cervical cancer arises rarely as a result of infection, with most infections being cleared by the host without clinical symptoms. Regression of anogenital warts is accompanied by a CD4+ T cell-dominated Th1 response, which is also seen in animal models [90,91,92,93,94,95]. In general, HPV infections evade both the adaptive and innate immune response, with the life cycle being totally intra-epithelial, without viraemia, cell lysis or inflammation. During persistent infection, pro-inflammatory cytokines are not released, and the signals for Langerhans cell and dendritic cell activation and recruitment are largely absent [96]. In fact, cells supporting viral late gene expression, and which may contain high levels of viral proteins, are shed from the surface of the epithelium away from immune surveillance. In general, a failure to develop an effective host immune response correlates with persistent infection and an increased probability of progression toward invasive cancer.

In HPV-associated neoplasias, the ordered expression of viral gene products necessary for virus synthesis does not occur, leading to a non-productive or abortive infection rather than a productive infection (Figure 4B). In this situation, the deregulated expression of the high-risk E7 protein can stimulate host genome instability through deregulation of the centrosome cycle, with the deregulated expression of the high-risk E6 protein contributing to the accumulation of cellular mutations as a result of inhibition or loss of E6 function. This deregulation and the subsequent effects on the cell can eventually lead to the development of cancer (reviewed in [2]).

## 5. Epithelial Stem Cells and Their Postulated Role in Papillomavirus-Associated Disease

Current thinking suggests that the development of papillomavirus-associated disease requires the infection not just of an epithelial basal cell, but more specifically an epithelial tissue stem cell at a pluristratified cutaneous or mucosal site [97,98,99]. In general, papillomavirus-associated lesions often persist for many years. Epithelial tissue renewal is achieved by proliferation of stem or stem-like cells in the basal layer, to produce transit amplifying cells that have a limited proliferative capacity, and which subsequently go on to terminally differentiate [100]. Once committed to differentiate, these cells are pushed towards the cell surface by the production of new cells beneath them, and are eventually lost from the epithelial surface as terminally differentiated squames. This movement comprises the “epidermal flow”, which occurs over a shorter time period than the life span of a papillomavirus infection [101]. Thus, we assume that papillomaviruses target basal cells with stem cell-like properties [97], or in the case of the high-risk papillomaviruses, they confer such properties on the cells that they infect. The idea that papillomaviruses generally reside in an epithelial stem cell following infection, is compatible with our understanding of latency and reactivation from latency [68], and the clonal origin of papillomas which was established several decades ago [102]. Indeed, studies on animal models have shown that CRPV primarily targets the bulge region of the hair follicle, where epithelial stem cells reside [103], and in cervical neoplasia, the epithelial reserve cells have long been considered the target cell from which cervical disease arises [99,104]. However, the cellular targets of infection have not yet been mapped more precisely, and this still remains a hypothesis, albeit a plausible one.

## 6. Local Epithelial Structure and Sites of Papillomavirus Infection

**Figure 5:**
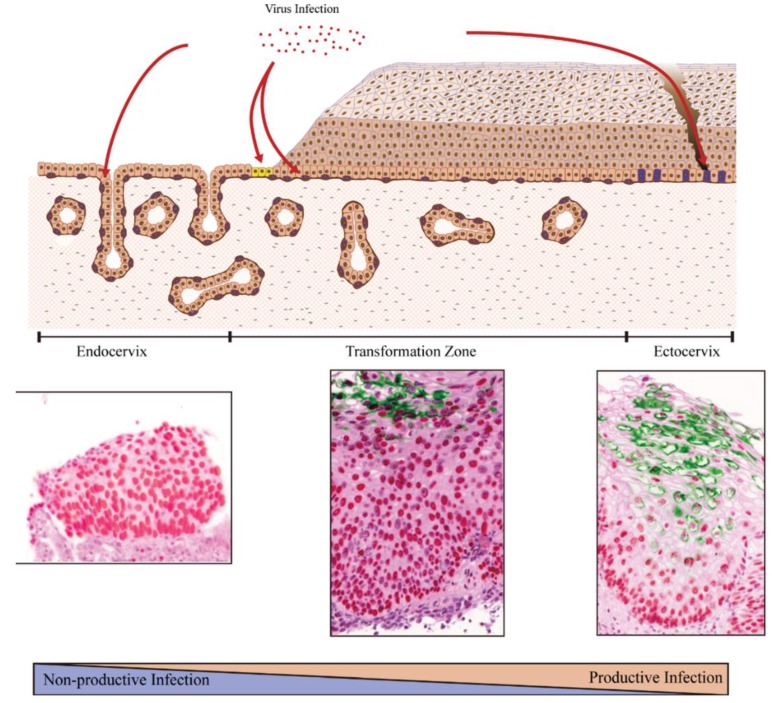
The difference of host cells (the site of infection) affects viral pathogenicity. Most cervical cancers arise at the cervical transformation zone. The transformation zone is maintained by a specialized type of tissue stem cell known as the reserve cell (shown in purple in the transformation zone), and possibly also by a cluster of cuboidal cells (**yellow**) localized more precisely at the squamo-columnar junction. These cells can maintain either the columnar epithelium of the endocervix or the stratified epithelium of transformation zone depending on their extracellular environment. In the ectocervix, the epithelium is populated by conventional epithelial tissue stem cells (purple in the ectocervix). The different characteristics of the various tissue stem cells that HPV infects are thought to influence the pattern of viral gene expression differently [41]. Current thinking suggests that productive infection is favoured at the ectocervix, while a non-productive or abortive infection is more likely at the endocervix. In the immunostains, MCM expression (**red**) indicates the expression of viral E7 protein. E4 expression (**green**) indicates the productive infection [59].

Epithelial tissue is composed of three components; epidermis, dermis and subepidermal tissue (subcutaneous tissue at skin sites). The epidermis is composed of multiple keratinocyte layers, and is the component that papillomaviruses target. In addition, the skin also consists of various functional appendages, including hair follicles, sebaceous glands, eccrine and apocrine sweat glands, finger and toe nails, as well as the inter-appendageal epidermis. Specialised epithelial sites contain additional appendages, such as the salivary glands of the oral cavity and the tonsillar crypts of the oropharynx. For papillomaviruses, these “specialist” structures represent particularly vulnerable sites, as they lack the highly structured barrier function usually associated with the epithelium. An additional level of complexity resides at transformation zone regions, where stratified epithelium abuts columnar epithelium, and it is generally accepted that high-risk HPV-associated neoplasias develop primarily at these sites [105]. High-risk HPV types are associated with the vast majority of cervical cancers (>99%), most of which arise at the cervical transformation zone, as well as the more than 90% of cancers that arise at the anal transformation zone. It is reasonable to suspect that transformation zones at other anatomical sites, such as at the boundary between the oesophagus and the stomach, may also be susceptible to HPV-associated neoplasia, but that these sites only rarely become infected because of their location. At the cervix, the transformation zone is maintained by a specialised type of cell known as the reserve cell [104], and possibly also by a cluster of cuboidal cells localised more precisely at the squamo-columnar junction [98]. Our current thinking suggests that these cell types represent the stem cells that maintain either the columnar epithelium of the endocervix, or the stratified epithelium of the transformation zone depending on their extracellular environment. These cells are thought to respond differently to signals from neighbouring epithelial cells and from the dermis, when compared to the more conventional epithelial stem cells that populate the stratified layers of the ectocervix. Indeed, our current model suggests that cervical neoplasia develops primarily at the squamo-columnar junction because these cells fail to properly regulate viral gene expression, leading to a non-productive or abortive infection rather than a productive infection (Figure 5) [59,106].

In this situation, deregulated viral gene expression facilitates the accumulation of genetic errors in the infected cell that no longer supports a productive infection (as described previously), leading eventually to the development of cancer.

**Figure 6:**
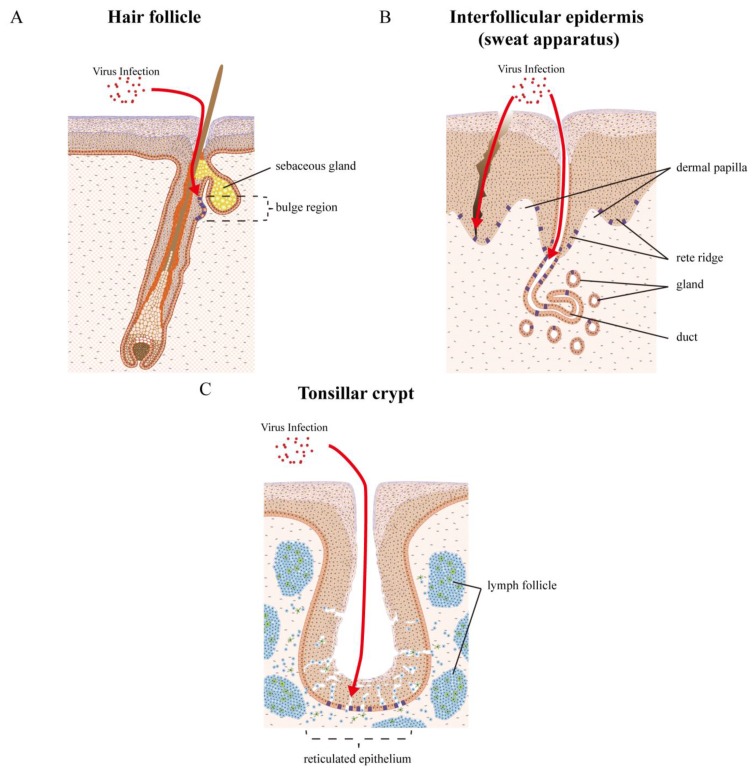
Epithelial tissue sites and tissue stem cells as targets for HPV infection. HPVs infect a variety of epithelial tissue sites, and can cause lesions in the vicinity of hair follicles, eccrine and apocrine sweat apparatus), nails, and also the inter-appendageal epidermis. Specialized epithelial sites contain other appendages, such as the salivary glands of the oral cavity and the tonsillar crypts of the oropharynx, where oropharyngeal cancers arise. The transformation zone regions, where stratified epithelium abuts columnar epithelium such as the cervical (Figure 5) or anal transformation zones, are other target sites where infection is thought to be facilitated. (**A**) The bulge region of the hair follicle is a well characterized region where the stem cells that populate cutaneous epithelial sites reside. HPV virions are thought to gain access to the epithelial stem cells (coloured purple), either through a wound or possibly through the hair follicle; (**B**) Between the hair follicles, the tissue stem cells are thought to reside in both rete ridge and over the dermal papilla, and are not thought to be clustered at any specific location in the basal component [109]. With the sweat apparatus, at least two distinct stem cell populations have been identified that may be accessible for infection, either in the gland or the duct. These are able to repair damaged epidermis. HPV virions are thought to gain access to these stem cells (coloured purple), either through a wound or maybe through the eccrine duct; (**C**) The tonsillar crypts are a highly specialized lymphoepithelial tissue. A dense lymphocyte infiltrate generally obscures the junction between the lymphoid and epithelial components and splinters the epithelial sheath into irregular nests and cords. This reticulated epithelium may facilitate viral access to tissue stem cells at an immune-privileged site, which can inhibit virus-specific T cell activity and thereby facilitate immune evasion during initial HPV infection and subsequent virus-induced malignant transformation.

By comparison with the cervical reserve cells, the stem cells that populate cutaneous epithelial sites are better characterised, and are known to reside in the bulge region of the hair follicle (Figure 6A) [107,108].

Because these cells can mediate the repair of damaged interfollicular epidermis, the interfollicular stem cells were for some time, thought to be supplied by follicular stem cells routinely [110]. However, recent work has shown that stem cells in the hair follicle do not contribute to epidermal homeostasis, but points instead to the presence of a distinct population of interfollicular epidermal stem cells [111]. This idea is supported by several observations [112], including the visualisation of discrete slow-cycling cells that are interspersed throughout the basal layer in mouse interfollicular epidermis [113] and in monkey palm epidermis [114], as well as the visualisation of clonal regenerating units of interfollicular epidermis (Figure 6B) [115]. In human skin, however, our inability to mark slow-cycling cells using *in situ* approaches, coupled with a lack of specific stem cell markers, has meant that the identification and location of interfollicular epidermal stem cells remains uncertain. Although it is generally accepted that eccrine sweat duct-derived cells can repair damaged epidermis [116], little attention has been paid to the sweat gland as an additional location of epidermal stem cells. Recently, stem cell populations in sweat glands and ducts have however been identified by lineage-tracing (Figure 6B) [117].

## 7. Examples of Papillomavirus Niche-Adaptation and Tropisms

Given the genotype-specific tropisms of papillomaviruses and the varied pathologies associated with infection, it is not unreasonable to think that different papillomaviruses have become well adapted to their respective epithelial niches. Although this has already been overviewed to some extent in the preceding paragraphs, the diversity of niche-adaptation can perhaps be appreciated by reference to particular examples that are outlined below.

Schmitt *et al.* [103] have shown that the primary target cells of CRPV co-localize with the hair follicle stem cells. In this study, E6 and E7 transcripts were detected at 11 days post-infection, despite the absence of apparent disease, with expression of these genes representing an early marker of infection. The subsequent finding that viable keratinocyte clones isolated from these infected follicles were CRPV mRNA-positive, suggests infection of a replication competent cell, which given the location, is suspected to be a bulge stem cell. A similar cellular target and epithelial tropism has also been suggested for the HPV types associated with epidermodysplasia verruciformis (EV). These HPV types usually come from the Genus Betapapillomavirus, and can be detected in plucked hair from different body sites. EV is a rare autosomal recessive genodermatosis associated with a high risk of skin carcinoma [11,118,119]. In many cases, the disease is linked to mutations in two genes (EVER1 and EVER2) that are expressed in the endoplasmic reticulum, and which form a complex with zinc-transporter-1 (ZnT-1), thereby controlling zinc balance [120]. Lesions generally start on the dorsa of the hands and the forehead, and spread progressively to the limb, neck and trunk. However, the mucous epithelium is not involved. EV skin lesions are hardly seen on the palmoplantar skin, even though they are frequently productive in nearby epithelium. At a clinical level, it is very clear that the Betapapillomavirus types associated with EV precisely “select” their preferential anatomical sites. The frequent detection of EV-PV in plucked hairs points to the hair follicle as an important site of infection for EV-PV [121,122].

**Figure 7:**
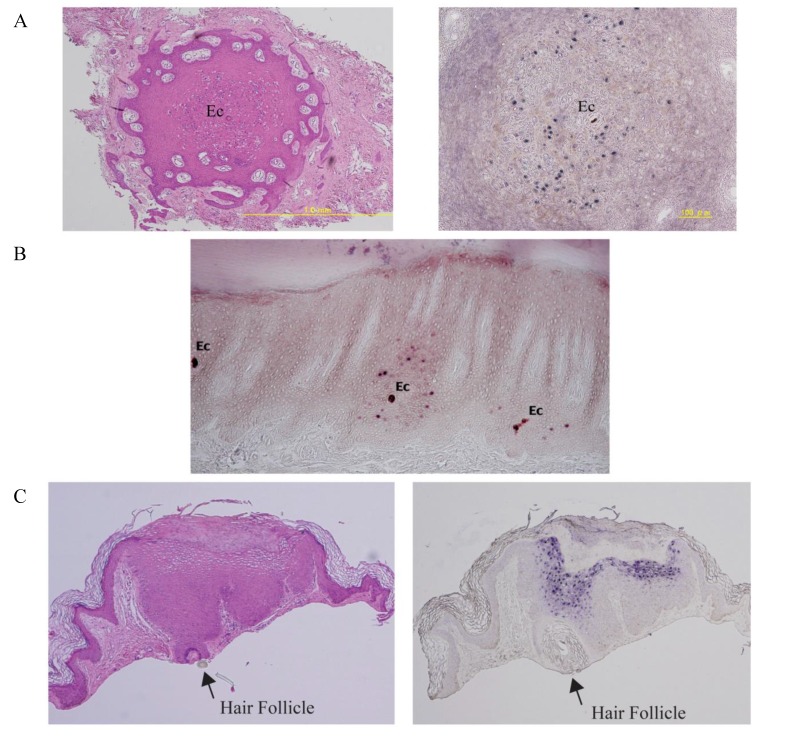
Examples for HPV targeting cutaneous appendages. (**A**) Haemotoxylin & eosin stain (**left**) of a horizontal section of a tiny wart. An eccrine (Ec)-centered distribution of histological changes is observed. HPV1 DNAs are identified within the pathology changes following DNA *in situ* hybridization (**right**); (**B**) HPV63 DNA is identified in resident keratinocytes in the vicinity of eccrine ducts (Ec) in a ridge of the plantar skin following DNA *in situ* hybridization; (**C**) HPV6/11 histopathological changes are identified in the resident keratinocytes in and around the hair follicle (arrow) by haemotoxylin & eosin staining (**left**) and DNA *in situ* hybridization (**right**).

HPV1 and 63 are members of the Genus Mupapillomavirus, and appear to target eccrine ducts in the palmoplantar skin. HPV1 causes typical myrmecia warts on the palms and soles [27,123], and it is quite rare for this HPV type to infect and cause lesions at extra-palmoplantar sites. The most characteristic histological feature is eosinophilic granular intracytoplasmic inclusion bodies (Gr-ICBs), which are larger and more numerous in the upper layer of the epidermis, and which are pathognomonic of infection by this virus. HPV1 infection provided a good example of how the site of infection influences clinical features and can affect viral gene expression [43]. Lesion development occurs on the skin surface ridges on the palms or soles. Histopathological studies and *in situ* hybridization analysis of early lesions have revealed that both HPV1-associated disease-pathology and *in situ* DNA-positivity are closely associated with eccrine ducts (Figure 7A, and our unpublished data).

HPV63, which is the second Mupapillomavirus type, induces tiny punctate warts (0.5 to 2 mm) characterised by the presence of heavily-stained tonofibril-like structures referred to as filamentous inclusion bodies [27,29]. To date, HPV63-induced warts have only been reported on the sole, with HPV63 DNA being detected both in keratinocytes around acrosyringiums (Figure 7B) and also in the uppermost portion of the eccrine dermal duct, which was hypertrophied with the HPV infection [124].

The final example focuses on HPV6 and 11, which share 85% sequence identity and belong to same HPV species (Alphapapillomavirus 2). Interestingly, in contrast to EV-HPVs from the Genus Betapapillomavirus, and which are both detectable in plucked pubic and eyebrow hairs, HPV6 and 11 could be detected only in hair plucked from pubic and perianal regions of patients with genital warts, but not in eyebrow hairs of the same patients. It has been suggested that HPV6 and 11 have a more limited epithelial distribution [125]. In contrast to this, Kocjan *et al.* [126], showed the presence of four genital HPVs (HPV6, 11 31 and 58) in plucked eyebrow hairs from immunocompetent individuals, suggesting that these viruses may sometimes be more widely distributed, but that they develop lesions preferentially at anogenital or oral sites. Since HPV6 and 11 are well-known causative agents for condyloma acuminatum, and can be detected in pubic and perianal hairs, a follicular distribution for this virus may exist. In fact, hair follicle-associated papules are sometimes observed in conjunction with the larger more prominent lesions at anogenital sites, with hair follicles being present widely at many epithelial sites. Interestingly, histological studies show that HPV6/11 DNA can also be detected in association with hair follicles as well as the vicinity of sweat apparatus or inter-appendageal epidermis, along with associated histological changes (Figure 7C, and our unpublished data). HPV6 and 11 are also causative agents of laryngeal papillomatosis, although in this instance the target cells remain unclear. The vocal cord, where HPV6 and 11 can also produce papillomatosis consists of squamous epithelium, pseudostratified epithelium and a transitional zone, which may facilitate access to stem-like cells at this site.

## 8. High-Risk Mucosal HPV Types

The high-risk mucosal HPVs, such as HPV16, 18, 31 and 33 appear to have evolved a number of additional characteristics that are not shared by low-risk papillomaviruses. These relate primarily to the function of the E5, E6 and E7 gene products, and the regulatory mechanisms that govern their expression (reviewed in [2]). Many functional studies have however focused on the contribution of these viral proteins to cancer development rather than life-cycle completion, and a detailed comparison of the role of these proteins in the life cycles of high and low-risk papillomaviruses remains to be done. A prominent characteristic of high-risk E6 proteins is the presence of a C-terminal PDZ-binding motif [127,128,129,130,131], along with an ability to bind and degrade the cellular p53 protein [132,133,134]. In addition, the high-risk HPV types have been shown to express their E6 and E7 proteins from a single promoter, with the levels of these two proteins being regulated by differential splicing [1,135]. This contrasts with the presence of separate promoters that regulate E6 and E7 expression in low-risk HPV types, such as those from the Alphpapillomavirus genus. Although there are additional key differences between these broad groups of papillomaviruses, these properties in themselves are known to have a major impact on high-risk virus pathogenesis. Although the precise advantage that the high-risk HPV types gain from these additional functions remains unclear, it is very likely to be related to particular sites of infection and their different strategies for productive infection at these sites. Clearly, the high-risk viruses have an ability to drive cell cycle entry as well as cell proliferation, which may be an important part of their normal life cycle at mucosal sites [83]. High-risk HPV-associated cancers occur predominantly within the cervical and anal transformation zone, as well as in the crypts of the oropharynx (Figure 6C) [136], which are thought to be sites where viral gene expression is poorly controlled. Given the preferential tissue distributions of different papillomaviruses, it is not surprising that particular HPV types may sometimes infect sites where full productive infection is either not supported or supported only poorly. The different cancer risk at the penis, vagina and vulva, when compared to the cervix or anus, illustrates this well [137]. In addition, the different risk of cancer progression associated with different HPV types (e.g., HPV16, 18 and 45) must reflect the different basic tropisms or tissue-specificities of the species Alphapapillomavirus 7 and Alphapapillomaviru 9 [138], and the different ways in which their genes are expressed and achieve their effects at these different epithelial sites. In addition to infection at these genital sites, some high-risk types can be found at other cutaneous sites where they cause “Bowenoid papulosis” [139,140].

## 9. Conclusions

Papillomaviruses have evolved over many millions of years to propagate themselves in a range of different species including humans. The evolution of skin glands, hair follicles and other ecological niches has provided papillomaviruses with new opportunities for infection. These have been followed by niche-adaptation, and it is thought that through this route, the great diversity of papillomaviruses has arisen. In general, β- and Gammapapillomavirus types have evolved a strategy of causing chronic asymptomatic infections accompanied by long-term virion-production and shedding from infected epidermis. This strategy minimises the risk of immune clearance, and depends on a balance between immune suppression and virus synthesis without any marked effect on host-fitness. By contrast, other papillomaviruses, such as those from the Genus Alphapapillomavirus, have evolved an array of sophisticated immune evasion strategies that allow them to cause prominent and persistent papillomas, even in immune competent individuals. Immune clearance of such lesions can however lead to asymptomatic or latent infections, with the possibility of an increase in viral copy number upon immunosuppression. Thus, both the cellular environment and the site of infection are important determinants of viral gene expression and virus activity. HPV pathogenicity, however, also reflects differences in viral gene function, which is evidenced most significantly when high and low-risk HPV types are compared. The genotype-specific association with disease, and more specifically with neoplasia, provides the basis for the recent introduction of DNA testing in cervical screening, and underlies the introduction of HPV 16/18 vaccines to protect against cervical cancer. High-risk mucosal HPVs do however have different cancer associations at different sites, prompting a detailed analysis of site-specific patterns of gene expression and gene function at different epithelial sites, such as the endocervix, the cervical transformation zone and the ectocervix, as well as the tonsillar crypts and other oropharyngeal sites where HPV-associated neoplasia can develop. An understanding of the site-specific aspects of HPV infection should facilitate the development of better strategies for disease treatment (e.g., target antivirals, immunotherapeutics) and disease management (e.g., risk assessment), and is very likely to provide new insight into epithelial biology and immunology.
